# Supplementary material for: Incidence of loiasis clinical manifestations in a rural area of the Republic of Congo: Results from a longitudinal prospective study (the MorLo project)
Source: PLoS Negl Trop Dis. 2025 Feb 12;19(2):e0012868. doi: 10.1371/journal.pntd.0012868 (PMC11844906; doi:10.1371/journal.pntd.0012868)
Supplement: S1 File — (PDF) [file pntd.0012868.s001.pdf]

ID du patient : ………………… Semaine du __/__/____au __/__/____

☐ Janvier	☐ Février	☐ Mars	☐ Avril	☐ Mai	☐ Juin	☐ Juillet	☐ Août	☐ Septembre	☐ Octobre	☐ Novembre	☐ Décembre

☐ A. Ver de l'œil

Entourer les jours où cela s'est produit

1	2	3	4	5	6	7	8	9	10	11	12	13	14	15	16
17	18	19	20	21	22	23	24	25	26	27	28	29	30	31	

A1. Avez-vous eu mal ? ☐ 0 – Non ☐ 1 – Un peu ☐ 2 – Moyennement ☐ 3 – Beaucoup

A2. Si vous avez eu mal, la douleur apparaissait : ☐ 0 – Rarement ☐ 1 – Souvent ☐ 2 – Tout le temps

A3. Est-ce que cela vous a empêché de dormir ? ☐ 0 – Non ☐ 1 – Oui

A4. Est-ce que cela vous a gratté ? ☐ 0 – Non ☐ 1 – Oui, un peu ☐ 2 – Oui, beaucoup

A5. Avez-vous pris un traitement ? ☐ 0 – Non ☐ 1 – Oui : lequel ? _________________

☐ B. Œdème (chaleur, gonflement)

Entourer les jours où cela s'est produit

1	2	3	4	5	6	7	8	9	10	11	12	13	14	15	16
17	18	19	20	21	22	23	24	25	26	27	28	29	30	31	

B1. A quel(s) endroit(s) ? ____

B2. Avez-vous eu mal ? ☐ 0 – Non ☐ 1 – Un peu ☐ 2 – Moyennement ☐ 3 – Beaucoup

B3. Si vous avez eu mal, la douleur apparaissait : ☐ 0 – Rarement ☐ 1 – Souvent ☐ 2 – Tout le temps

B4. Est-ce que cela vous a gratté ? ☐ 0 – Non ☐ 1 – Oui, un peu ☐ 2 – Oui, beaucoup

B5. Est-ce que cela vous a empêché de dormir ? ☐ 0 – Non ☐ 1 – Oui

B6. Avez-vous pris un traitement ? ☐ 0 – Non ☐ 1 – Oui : lequel ? _________________

☐ C. Douleur aux articulations

Entourer les jours où cela s'est produit

1	2	3	4	5	6	7	8	9	10	11	12	13	14	15	16
17	18	19	20	21	22	23	24	25	26	27	28	29	30	31	

C1. A quel(s) endroit(s) ? ____

C2. Comment était la douleur ? ☐ 1 – Faible ☐ 2 – Moyenne ☐ 3 – Forte

C3. La douleur apparaissait : ☐ 0 – Rarement ☐ 1 – Souvent ☐ 2 – Tout le temps

C4. Est-ce que cela vous a empêché de dormir ? ☐ 0 – Non ☐ 1 – Oui

C5. Avez-vous pris un traitement ? ☐ 0 – Non ☐ 1 – Oui : lequel ? _________________

☐ D. Démangeaisons, grattage

Entourer les jours où cela s'est produit :

1	2	3	4	5	6	7	8	9	10	11	12	13	14	15	16
17	18	19	20	21	22	23	24	25	26	27	28	29	30	31	

D1. A quel(s) endroit(s) ? ☐ 0 – Partout ☐ 1 – A certains endroits : lequel(s) ? ____________________

D2. Vous vous grattiez : ☐ 1 – Un peu ☐ 2 – Moyennement ☐ 3 – Beaucoup

D3. Avez-vous eu mal ? ☐ 0 – Non ☐ 1 – Un peu ☐ 2 – Moyennement ☐ 3 – Beaucoup

D4. Est-ce que cela vous a empêché de dormir ? ☐ 0 – Non ☐ 1 – Oui

D5. Avez-vous pris un traitement ? ☐ 0 – Non ☐ 1 – Oui : lequel ? _________________

☐ E. Absence au travail ou au champ (ou sortir dehors pour les vieux papa/maman)

Entourer les jours où cela s'est produit :

1	2	3	4	5	6	7	8	9	10	11	12	13	14	15	16
17	18	19	20	21	22	23	24	25	26	27	28	29	30	31	

E1. Pourquoi ? ☐ 1 – La douleur ☐ 2 – La fatigue ☐ 3 – La maladie

☐ 4 – Pour des raisons familiales ☐ 5 – Pour d'autres raisons : ____________________________________
