# Supplementary material for: Incidence of loiasis clinical manifestations in a rural area of the Republic of Congo: Results from a longitudinal prospective study (the MorLo project)
Source: PLoS Negl Trop Dis. 2025 Feb 12;19(2):e0012868. doi: 10.1371/journal.pntd.0012868 (PMC11844906; doi:10.1371/journal.pntd.0012868)
Supplement: S2 File — (PDF) [file pntd.0012868.s002.pdf]

Patient ID: ………………… Week: from __/__/____to __/__/____

☐ January	☐ February	☐ March	☐ April	☐ May	☐ June	☐ July	☐ August	☐ September	☐ October	☐ November	☐ December

☐ A. Eyeworm

Circle the days on which this happened																
	1	2	3	4	5	6	7	8	9	10	11	12	13	14	15	16
	17	18	19	20	21	22	23	24	25	26	27	28	29	30	31	

A1. Did you feel any pain? ☐ 0 – No ☐ 1 – A little ☐ 2 – Moderately ☐ 3 – Much

A2. If you were in pain, the pain appeared: ☐ 0 – Rarely ☐ 1 – Often ☐ 2 – Always

A3. Did it keep you awake at night? ☐ 0 – No ☐ 1 – Yes

A4. Did that make you itch? ☐ 0 – No ☐ 1 – Yes, a little ☐ 2 – Yes, a lot

A5. Have you taken any medication? ☐ 0 – No ☐ 1 – Yes: which? ________________

☐ B. Oedema (heat, swelling)

Circle the days on which this happened																
	1	2	3	4	5	6	7	8	9	10	11	12	13	14	15	16
	17	18	19	20	21	22	23	24	25	26	27	28	29	30	31	

B1. Where? ____

B2. Did you feel any pain? ☐ 0 – No ☐ 1 – A little ☐ 2 – Moderately ☐ 3 – Much

B3. If you were in pain, the pain appeared: ☐ 0 – Rarely ☐ 1 – Often ☐ 2 – Always

B4. Did that make you itch? ☐ 0 – No ☐ 1 – Yes, a little ☐ 2 – Yes, a lot

B5. Did it keep you awake at night? ☐ 0 – No ☐ 1 – Yes

B6. Have you taken any medication? ☐ 0 – No ☐ 1 – Yes: which? ________________

☐ C. Joint pain, arthralgia

Circle the days on which this happened																
	1	2	3	4	5	6	7	8	9	10	11	12	13	14	15	16
	17	18	19	20	21	22	23	24	25	26	27	28	29	30	31	

C1. Where? ____

C2. How was the pain? ☐ 1 – Low ☐ 2 – Moderate ☐ 3 – High

C3. The pain appeared: ☐ 0 – Rarely ☐ 1 – Often ☐ 2 – Always

C4. Did it keep you awake at night? ☐ 0 – No ☐ 1 – Yes

C5. Have you taken any medication? ☐ 0 – No ☐ 1 – Yes: which? ________________

☐ D. Itching, scratching

Circle the days on which this happened																
	1	2	3	4	5	6	7	8	9	10	11	12	13	14	15	16
	17	18	19	20	21	22	23	24	25	26	27	28	29	30	31	

D1. Where? ☐ 0 – Everywhere ☐ 1 – In some places: which? __________________

D2. You were scratching: ☐ 1 – A bit ☐ 2 – Moderately ☐ 3 – A lot

D3. Did you feel any pain? ☐ 0 – No ☐ 1 – A little ☐ 2 – Moderately ☐ 3 – Much

D4. Did it keep you awake at night? ☐ 0 – No ☐ 1 – Yes

D5. Have you taken any medication? ☐ 0 – No ☐ 1 – Yes: which? ________________

☐ E. Not at work or in the field *(or just out for elderly)*

Circle the days on which this happened																
	1	2	3	4	5	6	7	8	9	10	11	12	13	14	15	16
	17	18	19	20	21	22	23	24	25	26	27	28	29	30	31	

E1. Why is this? ☐ 1 – Pain ☐ 2 – Fatigue ☐ 3 – Illness

☐ 4 – For family reasons ☐ 5 – For other reasons: __________________________________
