# Supplementary material for: Incidence of loiasis clinical manifestations in a rural area of the Republic of Congo: Results from a longitudinal prospective study (the MorLo project)
Source: PLoS Negl Trop Dis. 2025 Feb 12;19(2):e0012868. doi: 10.1371/journal.pntd.0012868 (PMC11844906; doi:10.1371/journal.pntd.0012868)
Supplement: S3 File — (DOCX) [file pntd.0012868.s003.docx]

| **N of individuals** | **Frequency** (%) | **N of EW** | **N of CS** | **N of arthralgia** | **N of pruritus** | **N of absences** |
| --- | --- | --- | --- | --- | --- | --- |
| 489 | 49.3 | 0 | 0 | 0 | 0 | 0 |
| 47 | 4.7 | 0 | 0 | 1 | 0 | 0 |
| 35 | 3.5 | 0 | 0 | 0 | 1 | 0 |
| 27 | 2.7 | 0 | 0 | 1 | 0 | 1 |
| 19 | 1.9 | 0 | 0 | 1 | 1 | 0 |
| 18 | 1.8 | 1 | 0 | 0 | 0 | 0 |
| 17 | 1.7 | 0 | 0 | 2 | 0 | 0 |
| 17 | 1.7 | 0 | 1 | 0 | 0 | 0 |
| 15 | 1.5 | 1 | 0 | 1 | 0 | 0 |
| 12 | 1.2 | 0 | 1 | 1 | 0 | 0 |
| 10 | 1 | 0 | 0 | 0 | 2 | 0 |
| 9 | 0.9 | 0 | 1 | 1 | 0 | 1 |
| 9 | 0.9 | 1 | 1 | 1 | 1 | 1 |
| 8 | 0.8 | 0 | 0 | 3 | 0 | 0 |
| 7 | 0.7 | 0 | 0 | 1 | 1 | 1 |
| 7 | 0.7 | 0 | 1 | 1 | 1 | 0 |
| 7 | 0.7 | 1 | 0 | 0 | 1 | 0 |
| 7 | 0.7 | 1 | 0 | 1 | 0 | 1 |
| 7 | 0.7 | 1 | 1 | 1 | 1 | 0 |
| 6 | 0.6 | 0 | 0 | 2 | 0 | 1 |
| 6 | 0.6 | 1 | 0 | 0 | 0 | 1 |
| 5 | 0.5 | 0 | 2 | 2 | 0 | 2 |
| 4 | 0.4 | 0 | 0 | 0 | 1 | 1 |
| 4 | 0.4 | 0 | 0 | 2 | 1 | 0 |
| 4 | 0.4 | 0 | 1 | 0 | 1 | 0 |
| 4 | 0.4 | 1 | 0 | 2 | 0 | 0 |
| 4 | 0.4 | 1 | 1 | 0 | 0 | 0 |
| 4 | 0.4 | 1 | 1 | 1 | 0 | 1 |
| 3 | 0.3 | 0 | 0 | 1 | 2 | 0 |
| 3 | 0.3 | 0 | 1 | 2 | 0 | 0 |
| 3 | 0.3 | 0 | 1 | 2 | 0 | 2 |
| 3 | 0.3 | 1 | 1 | 1 | 0 | 0 |
| 2 | 0.2 | 0 | 0 | 2 | 0 | 2 |
| 2 | 0.2 | 0 | 0 | 2 | 1 | 2 |
| 2 | 0.2 | 0 | 0 | 2 | 2 | 0 |
| 2 | 0.2 | 0 | 0 | 2 | 3 | 0 |
| 2 | 0.2 | 0 | 0 | 3 | 0 | 2 |
| 2 | 0.2 | 0 | 0 | 3 | 0 | 3 |
| 2 | 0.2 | 0 | 0 | 3 | 1 | 2 |
| 2 | 0.2 | 0 | 0 | 3 | 1 | 3 |
| 2 | 0.2 | 0 | 0 | 3 | 2 | 0 |
| 2 | 0.2 | 0 | 0 | 4 | 1 | 1 |
| 2 | 0.2 | 0 | 1 | 0 | 0 | 1 |
| 2 | 0.2 | 0 | 1 | 1 | 0 | 2 |
| 2 | 0.2 | 0 | 1 | 2 | 1 | 0 |
| 2 | 0.2 | 0 | 1 | 2 | 1 | 3 |
| 2 | 0.2 | 0 | 1 | 3 | 0 | 0 |
| 2 | 0.2 | 0 | 2 | 0 | 0 | 0 |
| 2 | 0.2 | 0 | 2 | 0 | 1 | 0 |
| 2 | 0.2 | 1 | 0 | 1 | 1 | 0 |
| 2 | 0.2 | 1 | 0 | 1 | 1 | 1 |
| 2 | 0.2 | 1 | 0 | 1 | 2 | 1 |
| 2 | 0.2 | 1 | 0 | 2 | 1 | 0 |
| 2 | 0.2 | 1 | 0 | 2 | 1 | 1 |
| 2 | 0.2 | 1 | 0 | 2 | 2 | 0 |
| 2 | 0.2 | 1 | 1 | 1 | 2 | 0 |
| 2 | 0.2 | 1 | 1 | 2 | 0 | 0 |
| 2 | 0.2 | 2 | 0 | 0 | 0 | 0 |
| 2 | 0.2 | 2 | 0 | 0 | 0 | 2 |
| 1 | 0.1 | 0 | 0 | 0 | 0 | 1 |
| 1 | 0.1 | 0 | 0 | 0 | 2 | 2 |
| 1 | 0.1 | 0 | 0 | 0 | 3 | 2 |
| 1 | 0.1 | 0 | 0 | 1 | 1 | 2 |
| 1 | 0.1 | 0 | 0 | 1 | 2 | 1 |
| 1 | 0.1 | 0 | 0 | 1 | 3 | 0 |
| 1 | 0.1 | 0 | 0 | 2 | 2 | 1 |
| 1 | 0.1 | 0 | 0 | 2 | 4 | 0 |
| 1 | 0.1 | 0 | 0 | 3 | 0 | 1 |
| 1 | 0.1 | 0 | 0 | 3 | 1 | 0 |
| 1 | 0.1 | 0 | 0 | 3 | 1 | 1 |
| 1 | 0.1 | 0 | 0 | 4 | 0 | 0 |
| 1 | 0.1 | 0 | 0 | 4 | 0 | 3 |
| 1 | 0.1 | 0 | 0 | 4 | 2 | 0 |
| 1 | 0.1 | 0 | 0 | 4 | 3 | 0 |
| 1 | 0.1 | 0 | 1 | 0 | 1 | 1 |
| 1 | 0.1 | 0 | 1 | 1 | 1 | 1 |
| 1 | 0.1 | 0 | 1 | 1 | 5 | 0 |
| 1 | 0.1 | 0 | 1 | 2 | 2 | 0 |
| 1 | 0.1 | 0 | 1 | 3 | 3 | 3 |
| 1 | 0.1 | 0 | 1 | 4 | 2 | 0 |
| 1 | 0.1 | 0 | 1 | 8 | 0 | 7 |
| 1 | 0.1 | 0 | 2 | 0 | 0 | 1 |
| 1 | 0.1 | 0 | 2 | 0 | 0 | 2 |
| 1 | 0.1 | 0 | 2 | 0 | 2 | 0 |
| 1 | 0.1 | 0 | 2 | 1 | 0 | 0 |
| 1 | 0.1 | 0 | 2 | 1 | 0 | 1 |
| 1 | 0.1 | 0 | 2 | 1 | 0 | 3 |
| 1 | 0.1 | 0 | 2 | 1 | 2 | 0 |
| 1 | 0.1 | 0 | 2 | 2 | 1 | 0 |
| 1 | 0.1 | 0 | 2 | 2 | 1 | 2 |
| 1 | 0.1 | 0 | 2 | 2 | 2 | 0 |
| 1 | 0.1 | 0 | 2 | 3 | 0 | 0 |
| 1 | 0.1 | 0 | 3 | 2 | 2 | 6 |
| 1 | 0.1 | 0 | 3 | 2 | 3 | 3 |
| 1 | 0.1 | 0 | 4 | 0 | 0 | 4 |
| 1 | 0.1 | 1 | 0 | 0 | 1 | 2 |
| 1 | 0.1 | 1 | 0 | 0 | 2 | 0 |
| 1 | 0.1 | 1 | 0 | 0 | 3 | 0 |
| 1 | 0.1 | 1 | 0 | 0 | 3 | 1 |
| 1 | 0.1 | 1 | 0 | 1 | 0 | 2 |
| 1 | 0.1 | 1 | 0 | 1 | 2 | 0 |
| 1 | 0.1 | 1 | 0 | 1 | 3 | 0 |
| 1 | 0.1 | 1 | 0 | 1 | 4 | 6 |
| 1 | 0.1 | 1 | 0 | 2 | 0 | 1 |
| 1 | 0.1 | 1 | 0 | 2 | 0 | 2 |
| 1 | 0.1 | 1 | 0 | 3 | 0 | 2 |
| 1 | 0.1 | 1 | 0 | 3 | 0 | 4 |
| 1 | 0.1 | 1 | 0 | 4 | 2 | 0 |
| 1 | 0.1 | 1 | 0 | 7 | 0 | 6 |
| 1 | 0.1 | 1 | 1 | 0 | 1 | 0 |
| 1 | 0.1 | 1 | 1 | 0 | 2 | 2 |
| 1 | 0.1 | 1 | 1 | 1 | 0 | 3 |
| 1 | 0.1 | 1 | 1 | 1 | 3 | 0 |
| 1 | 0.1 | 1 | 1 | 2 | 0 | 1 |
| 1 | 0.1 | 1 | 1 | 2 | 1 | 2 |
| 1 | 0.1 | 1 | 1 | 2 | 2 | 2 |
| 1 | 0.1 | 1 | 1 | 3 | 1 | 2 |
| 1 | 0.1 | 1 | 1 | 3 | 3 | 0 |
| 1 | 0.1 | 1 | 1 | 6 | 6 | 5 |
| 1 | 0.1 | 1 | 2 | 0 | 1 | 0 |
| 1 | 0.1 | 1 | 2 | 1 | 0 | 1 |
| 1 | 0.1 | 1 | 2 | 3 | 2 | 0 |
| 1 | 0.1 | 1 | 3 | 2 | 0 | 0 |
| 1 | 0.1 | 1 | 3 | 4 | 1 | 0 |
| 1 | 0.1 | 2 | 0 | 0 | 0 | 1 |
| 1 | 0.1 | 2 | 0 | 0 | 1 | 0 |
| 1 | 0.1 | 2 | 0 | 0 | 3 | 0 |
| 1 | 0.1 | 2 | 0 | 1 | 0 | 0 |
| 1 | 0.1 | 2 | 0 | 1 | 0 | 4 |
| 1 | 0.1 | 2 | 0 | 1 | 1 | 0 |
| 1 | 0.1 | 2 | 0 | 1 | 2 | 0 |
| 1 | 0.1 | 2 | 0 | 2 | 0 | 0 |
| 1 | 0.1 | 2 | 0 | 2 | 0 | 2 |
| 1 | 0.1 | 2 | 0 | 2 | 1 | 0 |
| 1 | 0.1 | 2 | 0 | 3 | 3 | 1 |
| 1 | 0.1 | 2 | 0 | 6 | 1 | 0 |
| 1 | 0.1 | 2 | 1 | 0 | 0 | 0 |
| 1 | 0.1 | 2 | 1 | 0 | 1 | 0 |
| 1 | 0.1 | 2 | 1 | 0 | 5 | 0 |
| 1 | 0.1 | 2 | 1 | 1 | 1 | 0 |
| 1 | 0.1 | 2 | 1 | 1 | 1 | 2 |
| 1 | 0.1 | 2 | 1 | 2 | 2 | 0 |
| 1 | 0.1 | 2 | 1 | 3 | 0 | 3 |
| 1 | 0.1 | 2 | 1 | 7 | 3 | 6 |
| 1 | 0.1 | 2 | 2 | 0 | 1 | 0 |
| 1 | 0.1 | 2 | 2 | 0 | 3 | 0 |
| 1 | 0.1 | 2 | 2 | 1 | 0 | 0 |
| 1 | 0.1 | 2 | 2 | 2 | 1 | 2 |
| 1 | 0.1 | 2 | 2 | 2 | 2 | 0 |
| 1 | 0.1 | 2 | 2 | 2 | 2 | 1 |
| 1 | 0.1 | 2 | 2 | 2 | 2 | 2 |
| 1 | 0.1 | 2 | 2 | 4 | 1 | 0 |
| 1 | 0.1 | 2 | 3 | 4 | 4 | 1 |
| 1 | 0.1 | 3 | 0 | 0 | 0 | 0 |
| 1 | 0.1 | 3 | 0 | 0 | 0 | 3 |
| 1 | 0.1 | 3 | 0 | 1 | 0 | 4 |
| 1 | 0.1 | 3 | 0 | 1 | 1 | 4 |
| 1 | 0.1 | 3 | 1 | 1 | 1 | 1 |
| 1 | 0.1 | 3 | 1 | 1 | 2 | 4 |
| 1 | 0.1 | 3 | 1 | 3 | 1 | 3 |
| 1 | 0.1 | 3 | 2 | 0 | 2 | 0 |
| 1 | 0.1 | 3 | 2 | 0 | 4 | 0 |
| 1 | 0.1 | 3 | 2 | 2 | 1 | 0 |
| 1 | 0.1 | 3 | 2 | 5 | 2 | 5 |
| 1 | 0.1 | 3 | 3 | 2 | 2 | 2 |
| 1 | 0.1 | 3 | 3 | 4 | 5 | 3 |
| 1 | 0.1 | 4 | 0 | 4 | 1 | 0 |
| 1 | 0.1 | 4 | 0 | 4 | 3 | 5 |
| 1 | 0.1 | 4 | 2 | 0 | 2 | 1 |
| 1 | 0.1 | 4 | 2 | 3 | 2 | 2 |
| 1 | 0.1 | 5 | 0 | 0 | 0 | 0 |
| 1 | 0.1 | 5 | 0 | 0 | 3 | 9 |
| 1 | 0.1 | 5 | 1 | 1 | 1 | 0 |
| 1 | 0.1 | 5 | 4 | 4 | 5 | 1 |
| 1 | 0.1 | 6 | 0 | 0 | 0 | 6 |
| 1 | 0.1 | 8 | 0 | 3 | 4 | 1 |

**Supplementary material 2. Different patterns of symptoms**
