# Supplementary material for: Incidence of loiasis clinical manifestations in a rural area of the Republic of Congo: Results from a longitudinal prospective study (the MorLo project)
Source: PLoS Negl Trop Dis. 2025 Feb 12;19(2):e0012868. doi: 10.1371/journal.pntd.0012868 (PMC11844906; doi:10.1371/journal.pntd.0012868)
Supplement: S4 File — (DOCX) [file pntd.0012868.s004.docx]

| Event | Category | Incidence rate (/1000 PY) | 95%CI |
| --- | --- | --- | --- |
| EW | <30 yo | 88.4 | 47.5-164.2 |
|  | 31-40 yo | 171.0 | 118.1-247.7 |
|  | 41-55 yo | 294.9 | 243.6-357.0 |
|  | 56-70 yo | 341.7 | 284.6-410.2 |
|  | >70 yo | 269.0 | 183.1-395.1 |
|  | F | 309.9 | 259.9-369.5 |
|  | M | 240.2 | 205.7-280.5 |
| CS | <30 yo | 150.3 | 93.4-241.8 |
|  | 31-40 yo | 189.4 | 133.2-269.3 |
|  | 41-55 yo | 179.6 | 140.6-229.5 |
|  | 56-70 yo | 273.2 | 222.7-335.2 |
|  | >70 yo | 248.1 | 166.3-370.2 |
|  | F | 264.8 | 218.9-320.3 |
|  | M | 183.1 | 153.3-218.7 |
| Arthralgia | <30 yo | 203.4 | 135.2-306.1 |
|  | 31-40 yo | 330.4 | 253.0-431.3 |
|  | 41-55 yo | 588.8 | 514.1-674.3 |
|  | 56-70 yo | 719.3 | 634.0-816.1 |
|  | >70 yo | 655.5 | 512.1-839.1 |
|  | F | 664.7 | 589.3-749.7 |
|  | M | 489.5 | 439.1-545.7 |
| Pruritus | <30 yo | 256.9 | 178.5-368.6 |
|  | 31-40 yo | 281.2 | 210.6-375.5 |
|  | 41-55 yo | 382.4 | 323.2-452.4 |
|  | 56-70 yo | 368.6 | 309.1-439.6 |
|  | >70 yo | 196.3 | 125.2-307.7 |
|  | F | 335.0 | 282.8-396.8 |
|  | M | 330.7 | 289.8-377.4 |
| Absence from work | <30 yo | 61.9 | 29.4-129.8 |
|  | 31-40 yo | 134.4 | 88.5-204.1 |
|  | 41-55 yo | 337.3 | 282.1-403.4 |
|  | 56-70 yo | 315.4 | 260.7-381.5 |
|  | >70 yo | 655.0 | 511.7-838.5 |
|  | F | 345.3 | 292.2-407.9 |
|  | M | 270.6 | 233.8-313.2 |

**Supplementary Material 4.** Incidence rates of the 5 symptoms according to the age and sex
