# Supplementary material for: Incidence of loiasis clinical manifestations in a rural area of the Republic of Congo: Results from a longitudinal prospective study (the MorLo project)
Source: PLoS Negl Trop Dis. 2025 Feb 12;19(2):e0012868. doi: 10.1371/journal.pntd.0012868 (PMC11844906; doi:10.1371/journal.pntd.0012868)
Supplement: Attachment — Submitted filename: Review Letter.docx [file pntd.0012868.s006.docx]

December the 18th, 2024

Shaden Kamhawi and Paul Brindley

Co-Editor-in-Chief

*PLOS Neglected Tropical Diseases*

Jong-Yil Chai

Section Editor

*PLOS Neglected Tropical Diseases*

Dear Editors,

We hereby submit the revised version of our manuscript entitled “**Incidence of loiasis clinical manifestations in a rural area of the Republic of Congo: results from a longitudinal prospective study (the MorLo project)”** (PNTD-D-24-01074). We thank the four reviewers and the executive editors for their positive comments and appreciation of the paper.

We addressed each of the comments with detailed responses. We hope our manuscript will now meet the standards for publication.

Sincerely yours,

Jérémy CAMPILLO

jeremy.campillo@ird.fr

911 avenue Agropolis, 34000, Montpellier, France

*Tel: +33 4 67 41 61 62*

**Reviewer #1.**

*This manuscript examines the incidence of eyeworm, Calabar swellings, arthralgia, and pruritus in a Loa loa-endemic region of Congo as part of a very comprehensive project examining the morbidity associated with loiasis. The authors argue, given the high incidence of these signs/symptoms, that there is a clear impact on the quality of life of those affected.*

*Using a number of tools and weekly collected data obtained through community health workers, the study team was able to document the incidence of the various Loa-specific and Loa-non-specific clinical manifestations of loiasis and perform associations with microfilaremia, eosinophilia, and renal disease.*

*The study provides very important information but several issues need to be addressed:*

*1)Because the regions of study are hypoendemic for onchocerciasis, it is possible that up to 20 percent are co-infected with O. volvulus (Ov). Thus, it would be important to know how Ov infection is contributing to the pruritus and/or arthralgia incidence. Serologic assessment (e.g. antibodies to Ov16) would be extremely helpful. At a minimum, the authors should put into their statistical models a potential confounder of up to 20% Ov prevalence to see if this changes the real incidence of these 2 non-specific clinical manifestations.*

*2)Similarly, the authors very likely know the soil-transmitted helminth (STH) infection status of the group. Again, as many of the non-specific manifestations could be related to STHs, having this information will be critical.*

*3)On a similar track, what about scabies, lice, other ectoparasites that are known to cause intense pruritus?*

**We thank the reviewer for their pertinent comments. We have added information about onchocerciasis and STH prevalences. Regarding scabies, lice, and other ectoparasites, we did not have information about their prevalences. We agree that these may exist in the study population and may lead to itching. However, there is no reason to believe that these parasites are more prevalent in the loiasis-infected population than in the loiasis-uninfected population. Therefore, we believe that our incidence rates between the two populations are valid. We also added STH infection as a covariable in our 5 frailty models.**

Method: “The participant’s past exposure to Onchocerca volvulus was assessed using an Ov16 Rapid Diagnostic Test (Biplex L. loa/Ov16 RDT; Drugs & Diagnostics for Tropical Diseases, San Diego, California). The Ov16 RDT detects antibodies to Ov16 antigen. Two skin snips were collected from each patient with positive Ov16 RDT using a 2 mm Holth-type corneoscleral punch and incubated in saline at room temperature for 24 hours. Emerged mfs were counted under a microscope, and the individuals’ O. volvulus MFD, expressed as mfs per snip, were calculated using the arithmetic mean of the two snips. Finally, the participants were offered the option to provide stool samples for STH screening. STH infections were identified through the microscopic examination of stool specimens. Participants were supplied with a 50-mL plastic stool container and instructed to collect a morning stool sample. The collected specimens were placed in cooling boxes and shipped to the laboratory within 6 hours. Upon arrival, the samples were either immediately processed or stored overnight at 6°C. Using the Kato-Katz method, a thick smear was prepared from each stool sample and these smears were examined under a microscope at 40× magnification.”

Results: Twenty-two individuals (2.2%) tested positive for Ov16 RDT and none of them had O. volvulus mfs in the skin snips. Out of 771 individuals (77.8%) who volunteered for stool examinations, 391 cases tested positive for STH eggs (50.7%). Among these, there were no cases of hookworm infection, 332 cases of A. lumbricoides infection (43.1%), and 207 cases of T. trichiura infection (26.8%), with 146 cases exhibiting co-infections (18.8%)

**Reviewer #2.**

*In this manuscript entitled “Incidence of loiasis clinical manifestations in a rural area of the Republic of Congo: results from a longitudinal prospective study (the MorLo project)”, the authors provided much-needed information about the incidence rates of diverse clinical manifestations of loiasis. The experimental design and data presentation are clearly and support to the conclusion. The English of this manuscript should be carefully polished. Please consider all issues and concerns mentioned in the comments and suggestions, which hopefully can help authors improve the study. Specifically, please consider the following points before further process:*

*Abstract*

*-The aim of the study should be clearly stated in the background section.*

*-In the background section, page 2 line 33: “L. loa” should be defined the first time they are used.*

*-In results section, page 2 line 45: should specify if incidence rated was statistically significant rather than just say 'a significant elevated'.*

**We made the requested modifications in the abstract.**

*Introduction*

*-Abbreviations made in the Abstract, should be redefined in the main text the first time they are used.*

*-It is better to rearrange clinical manifestations with logical order as follows:*

*1-“The most typical are “Calabar swellings” (CS), which are transient angioedemas, and the migration of adult L. loa under the eye conjunctiva, often referred to as “eye worm” (EW)” (page 4, lines 71-73)*

*2-“Regarding CS, about 40% of infected adults report having experienced this manifestation at least once in their lifetime. CS can manifest with localized or general pruritus and varying levels of pain” (page 4, lines 84-86)*

*3-“EW episodes are transient, lasting from a few hours up to 7 days, are associated with photophobia and ocular itching, inflammation, and pain, usually resolving without complications” (page 4, lines 82-84)*

*4-“Other unspecific general manifestations include asthenia, headaches, transient nerve palsies, arthralgia, myalgia, pruritus and skin rashes [8–10]. It is generally reported that these general symptoms are well known and attributed to loiasis in affected populations [11]. Loiasis is described as a major reason for medical consultations in highly endemic regions, but there is actually limited data on the prevalence of loiasis-associated manifestations, especially concerning those which are non-specific. Indeed, it is hard to attribute the latter solely to L. loa infection because they can be due to other causes (eg. arthralgia and myalgia can be due to fieldwork or pruritus to other parasitic infections).” (page 4, lines 82-84)*

**We made the requested modifications in the introduction.**

*Methods*

*-The ethical approval number was not stated.*

**It is stated in the ethics statement paragraph: N˚ 036/CIE/FCRM/2022.**

*-Minor changes:*

*•Page 5 line 104-107: Detete “To date, no mass treatment with ivermectin for onchocerciasis had been implemented, but deworming campaigns with albendazole, restricted to children, are regularly carried out to control soil-transmitted helminthiases.”*

**We would prefer to keep this sentence as it allows the reader to better understand the context of the study site.**

*•Page 5 line 101: redefine (yo)*

*•Page 5 line 101: redefine abbreviation of “microfilaremia” the first time it is used “mfs”*

*•Page 7 line 144: Don’t repeat “of” just the first time it is used in sentence.*

**We believe that all of this is already done in our manuscript.**

*Results*

*-Cut off point of statistically significant results should be clearly mentioned and stated in all tables (p value).*

*-Any statistically significant result should be specified “statistically significant” rather than just say “significant”.*

*-p in statistical analysis italic*

**All our statistical analysis requiring a p-value are showing it. We added “statistically” where it was needed. We followed PlosNTD author guidelines for p-value presentations.**

*Discussion*

*-The discussion is well organized and written. However, it is better to mention limitations of assessing pruritus “One of the limitations of assessing pruritus is that it can be isolated or associated with other symptoms. The episodes of pruritus reported concerned only isolated pruritus and not pruritus associated with other manifestations. associated with other manifestations. It is therefore likely that the prevalence and incidence of pruritus are underestimated.” (page 21 lines 308-309 and page 22 lines 310-311) “before “Therefore, such data should be interpreted with caution.” (page 21 line 304)*

*-Recommendations should be stated in conclusion.*

**We made the requested modifications. We have no recommendations at the moment concerning the management of those symptoms. Our findings should emphasize the impact of loiasis on quality of life.**

**Reviewer #3.**

*The authors report on a landmark project (MorLo) that sets out to better define the clinical characteristics of loiasis in patients of a high transmission region in the Republic of Congo. The study was diligently designed, carefully conducted, and the analysis of the incidence of common signs and symptoms described in this paper is highly important. Until today, we mostly rely on data from cross-sectional surveys about the incidence of common signs and symptoms whereas this study allowed for the first time to assess the incidence and duration of these signs and symptoms.*

*The main findings are a substantial population attributable fraction of absence from work (22%), arthralgia (13%), and pruritus (17%). Importantly, these common symptoms were associated with the history of “Eyeworm Migration”, which substantiates the previously proposed concept of “migratory” loiasis showing more often subjective signs and symptoms of loiasis than “non-migratory loiasis”. In addition, the analysis shows for the first time that migratory loiasis (with its associated clinical symptoms) is age dependent.*

*The authors are the leading experts in the field of human filariasis today, who have shaped with their work many of our current concepts in the understanding of the disease and this work substantially adds to their important contribution to better define the clinical impact of loiasis. The authors have to be commended for their work and the data are much awaited to be published by the interested scientific community. My comments are mostly concerning the way of data presentation and analysis as I would find a slightly different approach more intuitive for the understanding of the important outcome of this study – while at the same time fully appreciating that the way the authors have chosen to present their findings is justified. I hope that some of my thoughts may be taken up in a revised version to be included in the analysis and depiction of data.*

*Major comments*

*My main comment concerns the way the data are depicted and analysed. While there is no question at all about the validity of the data and analysis, my feeling is that a slightly different depiction would make it easier for the reader to understand key biological features of loiasis that have been so well defined by this project:*

*“EW incidence rate 195 was estimated at 329.4/1000 PY in the MF+ group and 231.5/1000 PY in the MF- group.“ This analysis and the main depiction of data in table 1 and the results section on incidence of signs and symptoms:*

*To me this sounds as if these are incidence rates for the entire population, which include L. loa infected and uninfected persons (with about half of the population not reporting any sign or symptoms of loiasis). While this is of interest to understand the overall burden of disease in this community, it would be (more) interesting for me to understand the incidence of EW, CS, arthralgia, pruritus, etc in the 1) L. loa infected individuals (to understand better the disease) and separately in the 2) uninfected individuals (to understand the background for unspecific signs and symptoms). When you depict the data for the total population, the incidence is influenced both by the prevalence of the disease in this community and the frequency of occurrence of symptoms in infected individuals. The results therefore have less external validity for other communities with different prevalence/transmission intensities.*

**Thank you very much for your interest in our paper and for your insightful comments. As you are aware, there is currently no definitive test to diagnose loiasis in individuals who do not present with microfilaremia. The only symptom that can be definitively associated with *Loa loa* infection is the presence of the eye worm. In all our incidence calculations, we have represented the four populations that we believe are most relevant: MF+/EW+ (loiasis with clinical symptoms and microfilaremia), MF+/EW- (loiasis with microfilaremia without clinical symptoms), MF-/EW+ (loiasis without microfilaremia with clinical symptoms; occult loiasis), and MF-/EW- (virtually representing non-infected individuals, “background noise”, although we cannot definitively exclude the presence of a *Loa loa* infection). For the symptom EW, it is not possible to reason in terms of clinical symptoms because it is very difficult to definitively associate the other symptoms of interest (pruritus, arthralgia, and even CS as these remain self-reported and may be difficult for local health workers to diagnose. Therefore, we believe it is more pertinent to remain with our two populations for EW: MF+ and MF-. Additionally, we have calculated the incidence for each symptom in the overall population. We hope this clarifies our approach and we appreciate your understanding.**

*To me it would make more sense to categorize persons in infected and uninfected individuals based on microfilaremia and history of EW as the two most specific markers for occult and microfilaremic loiasis (one may argue whether CS is specific enough or not and include or not this in the case definition) – and calculate the incidence of signs and symptoms for these groups separately. This would provide an estimate of the clinical features of loiasis in the infected patients versus the “background” incidence of symptoms (arthralgia, pruritus, absence from work) in the uninfected. These estimates would have more external validity for individual patients in other transmission settings.*

*In a way the authors have used this approach by analyzing the data according to EW and MF status, defining the categories EW+/MF+, EW-/MF-, etc. What I would suggest, is to add the combined category EW+/MF+, EW+/MF-, MF+/EW- into one category: Loa infected. This will then provide the incidence for infected versus un-infected persons.*

**We have added the Loa-infected group in Table 2.**

*Finally, the authors also evaluate the difference in the occurrence of signs and symptoms in patients with or without history of EW (and CS). The authors confirm the previous finding that patients with history of EW have a much higher clinical burden than patients with MF only (Veletzky et al 2022 PLoSNTD Distinct loiasis infection states and associated clinical and hematological manifestations in patients from Gabon – a study that analyzed these concept for the first time based on cross-sectional data and clearly requires to be discussed in this manuscript). The classification of “migratory” versus “non-migratory” loiasis has been proposed for these two categories of patients and it may be worthwhile for the authors to use or discuss this concept of classification which seems to aptly determine the clinical penetrance of loiasis.*

**We thank the reviewer for this very pertinent comment. We added this comparison in the discussion:** *“Our study found lower prevalence rates of EW and CS in the adult population of the Congo (18.7% and 17.3%, respectively) compared to the higher prevalence rates reported in a highly Loa-endemic area of rural Gabon (42.2% for EW and 36.0% for CS). This discrepancy may be attributed to differences in the endemicity of Loa loa or in the demographic characteristics of the study populations.*

*The frequencies of events in our study for the MF+/EW+, MF-/EW+, MF+/EW-, and MF-/EW- groups were similar to those reported in the Gabon study for CS and arthralgia. Regarding pruritus, we observed a higher incidence rate in the EW+ groups compared to the EW- groups, whereas the Gabon study did not find any significant difference. This difference could be due to variations in the reporting and perception of pruritus among the study populations or differences in the local healthcare practices and diagnostic criteria.*

*Additionally, our findings confirm the previous observation that patients with a history of EW have a much higher clinical burden than those with microfilaremia only. This supports the classification of "migratory" loiasis (MF+/EW+ and MF-/EW+) versus "non-migratory" loiasis (MF+/EW- and MF-/EW-), which effectively determines the clinical penetrance of loiasis. This classification could be beneficial in determining the clinical impact of loiasis and should be considered in future studies.”*

*“Microfilaremia was associated with occurrence of EW episodes: 241 compared to amicrofilaremic individuals, those with 1–7,999 mfs/mL and 8,000–20,000 242 mfs/mL were 1.50 (P = 0.005) and 1.71 (P = 0.011) more likely to have experienced an EW 243 episode.“*

*The understanding of this analysis is difficult for me. As it stands it seems to me that uninfected persons (EW-/MF-) were mixed with occult patients (EW+/MF-) in one group and compared to patients with different levels of MF (and therefore all infected individuals). This does not seem informative to me as analysis, as we actually want to understand whether occult loiasis patients have more or fewer episodes of EW than microfilaremic individuals. This is inherently difficult to analyse as we have no marker for occult disease other than the clinical signs (mostly EW). A way out of this conundrum would be to analyze the number of EW events in those with at least one EW in the MF- versus those with at least one EW in the category of varying levels of MF+. In the current version the non-infected persons (which are mixed with the occult patients) seem to skew the group of MF-.*

**Thank you for your valuable feedback and for highlighting this important point.**

**We used multiple failure analysis and frailty models to calculate the incidence rates of EW, CS, arthralgia, pruritus, and absence from work (AfW), and to analyze factors associated with the increased incidence of each symptom. These models utilize the entire population to evaluate the occurrence of symptoms within it. We did not mix groups inappropriately; rather, we focused on the general population living in a Loa-endemic area, regardless of their microfilarial status or other symptoms or conditions.**

**By using this model, we found that microfilaremia is indeed associated with the occurrence of EW episodes. This approach allows us to assess the overall impact of microfilaremia on the population, providing insights into the broader epidemiological patterns in endemic regions. This association is also clearly visible in Table 2.**

**We appreciate your suggestion to analyze the number of EW events in those with occult loiasis. This is a valuable perspective and could offer additional insights into the clinical burden of occult loiasis compared to microfilaremic individuals. However, our current analysis aims to address the overall population rather than specifically comparing occult loiasis patients to microfilaremic individuals. We plan to publish another manuscript focusing on characteristics of mf+ vs. occult loiasis patients in the future.**

*The association of individual signs&symptoms with each other seems to strongly confirm the concept of migratory loiasis being the main driver for clinical disease as opposed to microfilaraemia (Veletzky et al 2022 PLoSNTD as above). It may therefore be worth considering an analysis using the concept of migratory loiasis versus non-migratory loiasis versus non-infected persons.*

**We thank the reviewer once again for their valuable feedback. We have acknowledged the necessity of citing the referenced article and have added a section in the discussion addressing this (see above). The association of individual signs and symptoms with each other strongly supports the concept that migratory loiasis is the main driver of clinical disease, as opposed to microfilaremia (Veletzky et al., 2022, PLoSNTD). Therefore, it may be worth considering an analysis using the concept of migratory loiasis versus non-migratory loiasis versus non-infected persons. We prefer to maintain our groups as EW+/MF+, EW-/MF+, EW+/MF-, and EW-/MF-. However, we have also added the category "Loa-infected," which includes the three groups EW+/MF+, EW-/MF+, and EW+/MF-.**

*Minor comments:*

*The estimates for the epidemiology of loiasis refer to an outdated analysis from 2011. A newer estimate based on these data and the population growth in rural regions of Central Africa estimates 20.1 million people residing in high transmission regions (as opposed to 11 million) as discussed in reference 10.*

**We made the modification.**

*The authors define loiasis as persons with either eyeworm or Calabar swelling (line 175). I wonder whether microfilaremia was not regarded as criterion (eg microfilaremia without EW and without CS)? In addition, CS seems not so specific for loiasis as it may also be caused other infections (mansonellosis, etc), rheumatic diseases, etc. It would be good if the authors would comment on this consideration and why they chose the definition as such.*

**We thank the reviewer for this valuable feedback. It is indeed a mistake, we modified it for “EW+ and/or MF+).**

*Attributable fraction: it is understood that the attributable fraction of absence from work etc relies on the hypothesis that all other factors are equal between infected and un-infected persons. In reality this may not be the case with infected individuals being more likely to suffer from other infectious diseases, potentially lower socioeconomic status, different work envirnoments, etc. This cautionary thought could be mentioned more clearly for this analysis.*

**Thank you for your insightful comments regarding the attributable fraction analysis. We understand your concern about the potential influence of external factors such as other infectious diseases, socioeconomic status, and work environments on the population attributable fraction (PAF). However, the calculation of PAF is designed to isolate the effect of the variable of interest—in this case, loiasis—under an "ideal" scenario where the exposure does not exist. This method assumes that all other factors remain constant and are not influenced by the presence of loiasis. In our study population, we believe that there is no significant difference in the profiles of infected and uninfected individuals regarding other infectious diseases, socioeconomic status, and work environments. Our study population is relatively homogeneous, which supports the validity of our PAF calculations.**

**Reviewer #4.**

*Are the results clearly and completely presented? Table 1 is not fully visible on the provided version*

*Do the authors discuss how these data can be helpful to advance our understanding of the topic under study? see comment, I think the new data can be discussed more with regard to available literature.*

*The manuscript by Campillo et al reports on the incident rates of various loiasis associated symptoms. The data was collected prospectively during a long follow up period in a large patient cohort as part of a larger study.*

*The prospective data collection and the short intervals of visits by HCWs with the villagers are factors supporting the quality of the data. In general, the manuscript is well written and easy to follow.*

*The authors provide symptom incidences and their association with respective loiasis manifestations - with very interesting results. It seems that eyeworm occurrence, in contrary to microfilaremia, is well associated with symptom occurrence.*

*My major comment is that the here reported results from the Congo are aligned with similar findings that have been described in a cohort from Gabon (https://doi.org/10.1371/journal.pntd.0010793) but this is not discussed.*

*This actually supports the relevance and validity of the here reported data. I therefore suggest that the authors discuss their findings in regard with previous data, with special regard to similarities and also differences as well as possible conclusions that may be drawn from those findings.*

**Thank you for your valuable feedback and for highlighting this important point. We add some lines about Gabon in the discussion (see above, reviewer 3).**

*Also, the absence of work and socioeconomic impact of loiasis has been described and this could also be addressed to improve the discussion.*

**We added the following sentence after the PAF calculation for work absenteeism: “***The socioeconomic impact of loiasis, including its effect on work absenteeism, underscores the importance of addressing this disease to improve overall community health and productivity.”*

*Otherwise I have mostly minor comments, which should be addressed before publication and which are outlined below.*

*Minor:*

*Abstract line 46: “EW occurrence appears influenced by microfilaremia.” In what way? Adapt for clarity.*

**We made the following modification: “***EW occurrence is more frequent when microfilaremia is present.”*

*Line 58: “Microfilaremia influenced EW occurrence, and CS correlated with EW, pruritus, and absence.“ Influence in what way? Absence of work? Adapt for clarification.*

**We made the following modification: “***EW occurrence is more frequent in individuals with microfilaremia. Absences from work are more frequent in those with a significant history of EW or arthralgia. CS occurrences are more frequent in individuals with a significant history of pruritus, arthralgia, or EW.”*

*Line 59: “These 59 findings emphasize the impact of loiasis on quality of life.” As the study is not aiming to address the quality of life (by e.g. using a QALY questionnaire) I suggest to rephrase this final sentence.*

**We remove this sentence.**

*Line 105: “To date, no mass treatment with 105 ivermectin for onchocerciasis had been implemented,..” I think “…has been… “ would be correct.*

**Done***.*

*Line 173: “This represents the 173 proportion of these symptoms that could potentially be eradicated if loiasis was eliminated.” Maybe “…potentially be avoided if…” is more adequate?*

**Done***.*

*Table 1: in the PDF the table is not fully visible.*

**It’s working in the Word format.**

*-Line for associated pruritus and CS the % should be 76.4 and 23.6 ?*

**Done***.*

*-Line for isolated pruritus intensity the % numbers don’t add up? (29.4+30.3+37.9= 97,6)*

**Done***.*

*Line 286- 288: “This study allowed us to estimate, for the first time, the incidence rates of the main clinical manifestations of loiasis in an endemic area and to assess the factors contributing to their occurrence.” Estimates have been done before but you have primary data and you literally counted the episodes - so I think you should clearly state that and may write “This study allowed us for the first time to prospectively count the incidence rates of the main clinical manifestations of loiasis in an endemic area and to evaluate the factors contributing to their occurrence.”*

**Done***.*

*Line 291 : citation 12 reported these eyeworm durations.*

**We added it.**

*Line 304-306: “Lastly, 305 treatment was taken in 42% of cases for arthralgia, around 30% for CS and pruritus and 23% 306 for EW. Treatment will be analyzed in a specific article.“ Is this similar to another article from Gabon (doi: 10.1371/journal.pntd.0012389)? Would be interesting to compare the results.*

**We've decided just to give these figures without going any further, as an article will be dedicated to them later on.**

*Line 317 - 319: “Regarding specific events, microfilaremia did not appear to significantly impact incidence rates 318 of CS, arthralgia, pruritus and AfW. In contrast, the occurrence of an episode of EW during 319 follow-up was strongly associated with the occurrence of CS, pruritus, and arthralgia episodes.” I suggest to discuss this in the light with previous reports. See comment above.*

**It has been done.**

*Line 359 – 363: The prospective design of this study, with a relatively long (13 months) follow-up period, the inclusion of nearly one thousand individuals, and the close weekly monitoring of the subjects are notable strengths. Additionally, the use of adapted statistical methods for repeated events with high unmeasured heterogeneity and discontinuous risk intervals are some of the strengths of this study.” “Of this study” is used twice, I suggest to rephrase.*

**Done.**

*Line 378-380: “To our knowledge, this study is the first to report incidence rates of the main clinical manifestations of loiasis in an endemic population.” See comment above.*

***Done.***
